# CT pulmonary angiography: an over-utilized imaging modality in hospitalized patients with suspected pulmonary embolism

**DOI:** 10.3402/jchimp.v3i1.20240

**Published:** 2013-04-17

**Authors:** Penchala S. Mittadodla, Sunil Kumar, Erin Smith, Madhu Badireddy, Mohamed Turki, Gloria T. Fioravanti

**Affiliations:** 1 Department of Internal Medicine, St Luke's Hospital, Bethlehem, PA, USA; 2 Department of Family Medicine, Neshoba General Hospital, Philadelphia, MS, USA; 3 Pulmonary and Critical Care Medicine, St Luke's Hospital, Bethlehem, PA, USA

**Keywords:** acute pulmonary embolism, PE, diagnosis of PE, CT pulmonary angiography, CTPA over utilization

## Abstract

**Aims:**

To determine if computed tomographic pulmonary angiography (CTPA) was overemployed in the evaluation of hospitalized patients with suspected acute pulmonary embolism (PE).

**Methods:**

Data were gathered retrospectively on hospitalized patients (n=185) who had CTPA for suspected PE between June and August 2009 at our institution.

**Results:**

CTPA was done in 185 hospitalized patients to diagnose acute PE based on clinical suspicion. Of these, 30 (16.2%) patients were tested positive for acute PE on CTPA. The Well's pretest probability for PE was low, moderate, and high in 77 (41.6%), 83 (44.9%), and 25 (13.5%) patients, respectively. Out of the 30 PE-positive patients, pretest probability was low in 2 (6.6%), moderate in 20 (66.7%), and high in 8 (26.6%) (p=0.003). Modified Well's criteria applied to all patients in our study revealed 113 (61%) with low and 72 (39%) with high clinical pretest probability. When modified Well's criteria was applied to 30 PE-positive patients, 10 (33.3%) and 20 (66.6%) were found to have low and high pretest probability, respectively (p=0.006). D-dimer assay was done in 30 (16.2%) of the inpatients with suspected PE and all of them were found to have elevated levels. A lower extremity duplex ultrasound confirmed deep venous thrombosis in 17 (9.1%) of the patients with suspected PE, at least 1 week prior to having CTPA.

**Conclusion:**

Understanding the recommended guidelines, evidence-based literature, and current concepts in evaluation of patients with suspected acute PE will reduce unnecessary CTPA examinations.

Computed tomographic pulmonary angiography (CTPA) has emerged as a prominent imaging technique to investigate patients for suspected pulmonary embolism (PE; 1, 2). In 1980, 3 million CT scans were done in the United States, which increased to 70 million in 2007 (3, 4). Burge et al. looked at a New York State Statewide Planning and Research Cooperative System database with 24,871,131 patients and found that the number of PE diagnoses nearly doubled, from 2,590 in 1994 to 4,920 in 2004 but PE deaths did not vary significantly over time, from 157 in 1994 to 159 in 2004. This suggests that the increased use of CTPA with increased diagnosis of PE did not have a corresponding decline in mortality (5). CTPA can also provide a greater detail of other pathology in the chest causing the patient's symptoms, but it is not without its adverse effects due to radiation and contrast exposure. This study investigates if CTPA is being overemployed without appropriately understanding the current concepts in the evaluation of suspected PE.

## Materials and methods

A retrospective study was done examining a 3-month period (June–August) in 2009 at a 475-bedded hospital, with the institutional review board approval. Electronic medical records of all hospitalized patients who underwent a CTPA for suspected PE during this period were reviewed. Data on demographics, clinical presentation, components of Well's criteria (Table 1), along with simultaneous usage of other investigations, including D-dimer and lower extremity ultrasonography (USG), were collected and analyzed. The Well's criteria (Table 2) and modified Well's criteria (Table 3) were used (2).

**Table 1:** Well's criteria

Clinical symptoms of DVT (leg swelling, pain with palpation)	3.0
Other diagnosis less likely that PE	3.0
Heart rate>100	1.5
Immobilization (≥3 days) or surgery in previous 4 weeks	1.5
Previous DVT/PE	1.5
Hemoptysis	1.0
Malignancy	1.0

**Table 2:** Well's clinical pretest probability

Probability	Score
Low	≤2
Moderate	2–6
High	≥6

**Table 3:** Modified Well's clinical pretest probability

Probability	Score
PE unlikely	≤4
PE likely	>4

## Results

CTPA was done in 185 hospitalized patients to diagnose acute PE based on clinical suspicion. Of these, 30 (16.2%) patients were tested positive for acute PE on CTPA. The Well's pretest probability for PE was low, moderate, and high in 77 (41.6%), 83 (44.9%), and 25 (13.5%) patients, respectively. Out of the 30 PE-positive patients, pretest probability was low in 2 (6.6%), moderate in 20 (66.7%), and high in 8 (26.6%). Modified Well's criteria applied to all patients in our study revealed 113 (61%) with low and 72 (39%) with high clinical pretest probability. When modified Well's criteria were applied to PE-positive patients, 10 (33.3%) and 20 (66.6%) were found to have low and high pretest probability, respectively.

A chi-square test of general association was conducted to determine if there is a significant relationship between the frequency of PE status and Well's scoring categories for the entire sample (N=185). Results revealed a statistically significant association (p=0.003). A statistical analysis done on modified Well's pretest probability alone for acute PE revealed a sensitivity, specificity, positive predictive value and negative predictive value of 66.6, 66.4, 27.7 and 91.1%, respectively in our study population. D-dimer assay was done in 30 (16.2%) inpatients with suspected PE and all of them were found to have elevated levels. A lower extremity USG confirmed deep venous thrombosis (DVT) in 17 (9.1%) patients with suspected PE, at least 1 week prior to having CTPA. ventilation perfusion (VQ) scan was done in 36 hospitalized patients compared to 185 CTPA studies during this time period at our institution.

## Discussion

CTPA positivity quoted in literature ranges from 13 to 33% in suspected PE patients (6, 7). In our institution, we had 16% of patients tested positive for acute PE on CTPA, which is at the lower end of the prevalence range, suggesting that we are overemploying CTPA in this patient population. Poor patient selection secondary to non-adherence to the recommended guidelines for evaluation of PE, along with the easy availability of CTPA imaging most likely contributed to these results. CT is readily available round the clock, whereas our nuclear medicine department provides access for a VQ scan during working hours on weekdays. Also, physicians may be worried about the medico-legal implications of an undiagnosed PE, thus practicing defensive medicine.

The incidence of contrast-induced nephropathy after having CTPA in patients for suspected PE is up to 12% (8, 9). Brenner et al. estimated that 1.5–2.0% of incident cancers could be attributed to radiation exposure from CT scans in the United States (10). The radiation risk is considerably higher particularly in younger patients, women and all patients who have repeated examinations (11). Although manufacturers and radiologists are making every effort to reduce the CT radiation dose without affecting the clinical usefulness of the study, reduction in radiation risk could be achieved primarily by decreasing the number of unnecessary imaging with appropriate justification (12, 13). The radiation dose information can be collected and tracked and this information was provided to patients, clinicians, and radiologists before performing the imaging to understand ones risk of future complications. In patients with low pretest probability, it may be appropriate for the physicians to explain the patient's condition along with pros and cons of imaging. This informed decision making may help the patient choose to wait and watch before proceeding with CTPA, which in turn may minimize medico-legal complications and reduce some inappropriate imaging.

Usage of VQ scans in suspected PE with normal chest radiograph results in considerably lower radiation exposure with comparable negative predictive value to CTPA (14). The total effective radiation from CTPA is up to five times greater than that from VQ scan and the cost of CTPA is nearly twice that of a VQ scan (14). Anderson's study on 1,417 patients with high pretest probability (modified Well's score>4.5) did not observe mortality difference between CTPA and VQ scan groups, suggesting that VQ scan is a safer and viable alternative when studying patients for PE (1).

In patients with acute PE, lower extremity duplex USG is positive in 50% of patients with clinical signs and symptoms of DVT (16, 17). In patients with co-existing clinical DVT, duplex USG as an initial imaging test is often sufficient to confirm venous thromboembolism (VTE), precluding the need for further testing (18, 19). We believe 9.1% of patients with positive clinical DVT on lower extremity USG in our study could have avoided CTPA.

D-dimer assay is a very sensitive but non-specific screening test for VTE as it is elevated in many conditions (20). A study done by Rathbun et al. revealed that a normal D-dimer assay was found only in 10% of the hospitalized patients with clinically suspected PE and negative CTPA or VQ scan, suggesting its limited clinical utility in this group (21). It has been shown that a normal D-dimer assay in patients with low (18, 22) or intermediate (23) pretest probability safely eliminates the need for further diagnostic testing. The Well's criteria, pretest probability categorization, and utility of D-dimer were established based on the study that was done on patients presenting to emergency department (22). Christopher Study Investigators recommended the use of D-dimer assay and modified clinical pretest probability after analyzing 3,306 patients of which 82% were outpatients (2), but we studied hospitalized patients. Therefore, it is difficult to triage hospitalized patients with suspected PE for further imaging in low pretest probability groups in view of such a low specificity of D-dimer assay in this group. In Well's criteria, ‘alternative diagnosis less likely than PE’ has a high interobserver variability, placing the same patient in a different category depending on who calculates it (24).

We acknowledge the limitations of a retrospective study where data might not be completely obtainable but care was taken to gather all of the information at the time of study. To evaluate current practice at our institution, the study was designed to be in retrospective nature, in order to eliminate the Hawthorne effect. We used Well's and modified Well's scoring system in our study but not the revised Geneva scoring system, which only uses objective measures. This may have reduced the interobserver variability in assessing the pretest probability but evidence is lacking to prove that one scoring system is better than the other in hospitalized patients.

## Conclusion

We recommend the importance of adherence to clinical guidelines to avoid unnecessary CTPA and potential complications associated with radiation and contrast administration.
